# Notch1 signaling pathway promotes invasion, self-renewal and growth of glioma initiating cells via modulating chemokine system CXCL12/CXCR4

**DOI:** 10.1186/s13046-019-1319-4

**Published:** 2019-08-05

**Authors:** Li Yi, Xingchen Zhou, Tao Li, Peidong Liu, Long Hai, Luqing Tong, Haiwen Ma, Zhennan Tao, Yang Xie, Chen Zhang, Shengping Yu, Xuejun Yang

**Affiliations:** 1 Department of Neurosurgery, Tianjin Medical University General Hospital, Tianjin, 300052 China; 2 Laboratory of Neuro-Oncology, Tianjin Neurological Institute, Tianjin, 300052 China; 3 Department of Neurosurgery, The Second Affiliated Hospital of Bengbu Medical College, Anhui, 233000 China; 4 Department of Radiation Oncology, Henan Cancer Hospital, The Affiliated Cancer Hospital of Zhengzhou University, Henan, 450000 China; 5 Neuro-Oncology Department, The University of Texas MD Anderson Cancer Center, Houston, 77030 Texas USA

**Keywords:** Glioma initiating cells, Notch1, CXCR4, Invasion, Migration

## Abstract

**Background:**

Glioma initiating cells (GICs), also known as glioma stem cells (GSCs), play an important role in the progression and recurrence of glioblastoma multiforme (GBM) due to their potential for self-renewal, multiple differentiation and tumor initiation. In the recent years, Notch1 has been found to be overexpressed in GICs. However, the regulatory mechanism of Notch1 in the self-renewal and invasion ability of GICs remains unclear. This study aims to explore the effect of Notch pathway on self-renewal and invasion of GICs and the underlying mechanisms.

**Methods:**

Bioinformatic analysis and immunohistochemistry (IHC) were performed to evaluate the expression of Notch1 and Hes1 in GBM samples. Immunofluorescent (IF) staining was performed to observe the distribution of Notch1 and CXCR4 in GBM and GICs. Both pharmacological intervention and RNA interference were employed to investigate the role of Notch1 in GICs self-renewal, invasion and tumor growth in vitro or in vivo. The crosstalk effect of Notch1 and CXCL12/CXCR4 system on GIC self-renewal and invasion was explored by sphere formation assay, limiting dilution assay and Transwell assay. Western blots were used to verify the activation of Notch1/CXCR4/AKT pathway in self-renewal, invasion and tumor growth of GICs. Luciferase reporter assay was used to testify the potential binding site of Notch1 signaling and CXCR4. The orthotopic GICs implantations were established to analyze the role and the mechanism of Notch1 in glioma progression in vivo.

**Results:**

Notch1 signaling activity was elevated in GBM tissues. Notch1 and CXCR4 were both upregulated in GICs, compared to Notch1 positive glioma cells comprised a large proportion in the CD133+ glioma cell spheres, CXCR4 positive glioma cells which usually expressed Notch1 both and dispersed in the periphery of the sphere, only represent a small subset of CD133+ glioma cell spheres. Furthermore, downregulation of the Notch1 pathway by shRNA and MK0752 significantly inhibited the PI3K/AKT/mTOR signaling pathway via the decreased expression of CXCR4 in GICs, and weakened the self-renewal, invasion and tumor growth ability of GICs.

**Conclusions:**

These findings suggest that the cross-talk between Notch1 signaling and CXCL12/CXCR4 system could contribute to the self-renewal and invasion of GICs, and this discovery could help drive the design of more effective therapies in Notch1-targeted treatment of GBMs.

**Electronic supplementary material:**

The online version of this article (10.1186/s13046-019-1319-4) contains supplementary material, which is available to authorized users.

## Background

Glioblastoma multiforme (GBM), the most common primary malignancy of the brain, is an aggressive tumor with a poor prognosis. Due to its complex heterogeneity, plasticity and therapy resistance, GBM remains one of the most difficult tumors to treat, with a 5-year survival rate of only approximately 5.5% [1], despite the progress made in surgery combined with radiotherapy and chemotherapy in the last decade [2]. Glioma initiating cells (GICs) is a group of tumor cells with self-renewal, unlimited proliferation and multi-differentiation abilities, GICs are often located in the hypoxic niche, perivascular niche and invasive niche [3–5]. Many studies have shown that GICs are probably involved in the initiation, development, invasion and migration of tumors, as well as in resistance to chemotherapeutic agents, leading to tumor recurrence [6]. Therefore, a better definition of how GICs interact with their microenvironment would facilitate understanding the basic biology of the tumor and the design of more effective therapies.

In recent years, Notch1 has been found to be overexpressed in GICs, and the Notch pathway plays a vital role in the stem maintenance of glioma cells [7, 8]. The family of Notch receptors consist of heterodimeric transmembrane proteins intimately involved in the determination of cell fate. Up to now, four Notch receptors have been identified in humans (Notch1–4), with five corresponding ligands including Delta-like-1, Delta-like-3, Delta-like-4, Jagged-1, and Jagged-2. Activation of the Notch pathway requires that the receptor bind to the ligand and undergo protease hydrolysis to produce the Notch intracellular domain (NICD), which is released into the nucleus and go through a series of reactions to activate downstream HES and HEY, thus, the corresponding biological effects are produced. Hes1 is commonly used as a biomarker of Notch1 pathway activation [9, 10]. Depending on the cell types, Notch signaling can positively or negatively influence proliferation, differentiation, and apoptosis [9]. It was also demonstrated that Notch promoted radio-resistance in glioma stem cells [11]. Unfortunately, targeting developmental pathways such as Notch will most likely not give us the elusive “magic bullet”, as most of the trials on Notch inhibitors remain in clinical phases I and II [12], and will require to develop rational combinations. Such combinations will be made possible only through a better understanding of the crosstalk between Notch and other molecules that may play roles in GICs in specific malignancies [13].

CXCR4, a cell surface chemokine receptor, is implicated in the growth, invasion, angiogenesis and metastasis in a wide range of malignant tumors, including leukaemia, breast cancer and very recently in glioma [14–17]. Interestingly, CXCR4 has been shown to be selectively present on cancer initiating cells (CICs) derived from several solid tumors and they play a critical role in regulating carcinogenesis [18, 19]. The few studies that have looked into the role of CXCR4 in GICs, proposed that CXCR4 positive cells have the capability of developing not only tumorspheres in serum-free medium (SFM) supplemented with growth factors, but also the other cardinal features required for a GIC population [16, 20, 21]. Targeting CXCL12/CXCR4 autocrine/paracrine loop could inhibit the survival and proliferation of glioma stem cells and drive the migration of GSCs to neurogenic zone [22, 23]. Additionally, Notch signaling has been implied in mediating chemotaxis system CXCL12/CXCR4 in the metastasis renal cell carcinoma and multiple myeloma [24, 25]. However, the regulatory mechanism of Notch1 and its crosstalk with CXCL12/CXCR4 in GICs remains unclear. In this study, we highlighted that blocking Notch1 signaling by MK0752 (a γ-secretase inhibitor) or shRNA weakened the invasion and growth of GICs, these effects were reversed by the CXCR4 stimulating factor CXCL12. In addition, Notch1 signaling could enhance transcription of CXCR4 through directly targeting its promoter by RBPJ. Collectively, our findings confirmed that Notch1 promotes GICs invasion self-renewal and tumor growth through the AKT/mTOR pathway mediated by CXCL12/CXCR4 axis and thus provide evidence of a promising target for GBM management.

## Methods

### Cell lines and cell culture

U87 and U251 glioma cells were purchased from the Institute of Biochemistry and Cell Biology (Shanghai, China). Cells were cultured in DMEM containing 10% FBS (Gibco, USA). After MACS, CD133+ cells were cultured in stem cell medium (DMEM/F12 medium supplemented with 20 ng/ml EGF, 20 ng/ml bFGF, and B27 (1,50, Invitrogen, USA)). The tumorsphere can be observed on the second day.

### Magnetic activated cell sorting

CD133+ glioma cells were collected by CD133 Micro Bead Kit (Miltenyi, Germany) The tumorspheres were cultured for 7 days, centrifuged, removed, and digested with 0.25% Trypsin-EDTA. After 0.5 min, the complete medium was added to terminate the digestion. After centrifugation, the supernatant was removed, to further reduce the interference of serum, serum-free DMEM/F12 medium was added to make a single cell suspension. The cell concentration was adjusted to 1 × 10^5^ cells/μl, 300 μl of the cell suspension, 100 μl of FcR and 100 μl of CD133 beads were added to PBS buffer supplemented with 5% fetal bovine serum and 2 mM EDTA, thoroughly followed by incubation at 4 °Cin the refrigerator for 30 min. After installation of the magnetic separation column and iron, the prepared cells were added to the cell separation column with 500 μl buffer filtered 4 times to obtain unlabelled cells. Finally, to remove the iron from the magnetic separation column, 1 ml of buffer was used to flush the column, then, the obtained CD133+ labeled cells were cultured in stem cell medium.

### Lentiviral transfection

Lentiviral shRNA constructs were obtained from GeneChem Co., Ltd., China. shRNA sequences were as follows: GCATGGTGCCGAACCAATACA (NOTCH1-shRNA1), GGAGCATGTGTAACATCAACA (NOTCH1-shRNA2). Cells were transfected with the lentiviral particles, according to the manufacturer’s recommendations. After infection, stable cell clones expressing the shRNA constructs were isolated by selection with 5 μg/ml puromycin solution. Cells were collected for further experiments at 48 h after the transfection.

### Patient data analysis

Patient data and gene expression datasets were obtained from Oncomine Datasets Platform (https://www.oncomine.org/resource/login.html). Funrich software (http://www.funrich.org/) was used as the tool for making heatmap. Twenty-seven panels of GICs and 36 panels of non-glioma initiating cells (NGICs) were downloaded from Microarray data of GEO (Accession No. GSE23806). Box-plot, Kaplan–Meier analysis and the resulting survival curves were performed using GraphPad Prism (version 6.0). All cut-off values for separating high and low expression groups were determined by median expression value.

### Immunofluorescence analysis

An original method was applied to label proteins in the glioma tumorspheres. Tumorspheres were placed into a cell insert (Millipore, US) and fixed with 0.4% paraformaldehyde solution (Solarbio, China). Then, the tumorspheres were washed three times with PBS, and incubated with primary antibodies overnight at 4 °C. Alexa-Fluor 488 conjugated anti-rabbit secondary antibody (1:1000, Life Technologies, USA) and Alexa-Fluor 594 conjugate anti-mouse secondary antibody (1,1000, Life Technologies, USA) were used for fluorescent double-staining. DAPI solution (Solarbio, China) was employed to label cell nuclei.

### Immunohistochemistry staining

Paraffin embedded tumor tissues were sectioned at 6 μm, deparaffinized, and rehydrated. For antigen retrieval, sections were treated for 20 min at 95 °C with 10 mmol/L citrate buffer (pH 6.0) in a laboratory microwave oven and subsequently washed with PBS. For immunohistochemistry, after quenching the endogenous peroxidase activity and blocking with normal goat serum, sections were incubated sequentially with the primary antibodies at 4 °C overnight, the next day, after rewarming for 1 h, sections were incubated with secondary antibodies (ZSGB-Bio, China) for 1 h at 37 °C. Immunostaining was performed using two-steps detection kit (ZSGB-Bio, China), which resulted in a brown precipitate at the antigen site. Subsequently, sections were counterstained with Mayer Haematoxylin solution (ZSGB-Bio, China) and mounted in mounting medium. The primary antibody was omitted for the negative controls. After dehydration, sections were examined using a light microscope. Protein expression levels were quantified on the basis of a multiplicative index of the staining extent (0–3) and the average staining intensity (0–3). The staining score is the product of staining extent and the staining intensity.

### In vitro migration and invasion assays

Cells (5 × 10^5^) were plated on the top side of polycarbonate Transwell filters (for migration assay) or on the top side of polycarbonate Transwell filters coated with Matrigel (for invasion assay) in the top chamber of a 24-well cell invasion assay (Coning, USA). Dissociated GICs were seeded into chambers, the cells were suspended in medium without serum, and medium supplemented with stem cell culture medium (20 ng/ml EGF, 20 ng/ml bFGF, and 1,50 B27) was used as a chemoattractant in the bottom chamber. The cells were incubated at 37 °C for 24 h (for migration assay) or 48 h (for invasion assay). The non-migratory or non-invasive cells in the top chambers were removed with cotton swabs. The cells that migrated and invaded the lower membrane surface were fixed in 4% paraformaldehyde for 10 min, air-dried, then stained crystal violet staining solution (Solarbio, China) and counted under a microscope at a 10 × magnification.

### Western blotting analysis

Cells were lysed in RIPA buffer (Solarbio, China) containing PMSF (dilution, 1:100; Solarbio, China), and proteins were extracted from cells cultured in the absence of FBS. Before denaturation, total protein concentration was determined using BCA protein assay kit (Solarbio, China), according to the manufacturer’s instructions. The extracted proteins were separated by performing sodium dodecyl sulfate-polyacrylamide gel electrophoresis (SDSPAGE), blotted onto PVDF membranes (Millipore, USA), and incubated overnight at 4 °C with the indicated primary antibodies: Anti-Notch1 (Abcam, 1:1000), Anti-NICD (Abcam, 1:1000), Anti-Hes1 (Abcam, 1:1000), Anti-CXCR4 (Abcam, 1:1000), Anti-Akt (cell signaling, 1:1000), Anti-pAkt (cell signaling, 1:1000), Anti-mTOR (cell signaling, 1:1000), Anti-pmTOR (cell signaling, 1:1000). Next, the membranes were washed and incubated with goat anti-rabbit/mouse IgG secondary antibody (dilution, 1:2000) for 1 h at room temperature. Protein expression was analyzed using GBOX (Syngene Company, UK) and a chemiluminescent HRP substrate (Millipore, USA).

### Tumorsphere formation assay and in vitro limiting dilution assay

GICs were plated in 96-well plates at a density of 1,000 cells per well and tumorsphere numbers and sizes were calculated at the seventh day after cell placement. For in vitro limiting dilution assay, GICs were implanted into a 96-well plate at a gradient of 20, 40, 60, 80, 100,120 or 140 cells per well, with 10 replicates for each gradient. The number of tumorsphere in each well was determined after incubation for 7 days, and the sphere formation efficiency were calculated using the Extreme Limiting Dilution Analysis (http://bioinf.wehi.edu.au/software/elda).

### Transfection and luciferase reporter assay

The binding site between RBPJ and CXCR4 promoter was predicted through promo online tool (http://alggen.lsi.upc.es/). Transfection of cells and luciferase assays were performed as described (Picard et al., 2012). Cells were transfected with the pGL3-based constructs containing the CXCR4 promoter plus the Renilla luciferase plasmid (pRL-TK). Then, the cells were harvested after 48 h for firefly/Renilla luciferase assays using the Dual-Luciferase Reporter Assay System (Promega). Luciferase activities were normalized to the co-transfected pRL-TK plasmid (mean ± SD).

### Intracranial xenografting of U87GICs

Animal experiments were approved by the Ethical Committee of Tianjin Medical University General Hospital. U87GICs with scrambled lentivirus and Notch1 shRNA lentivirus were respectively co-infected with a luciferase-expressing lentivirus. Mice were anaesthetized, placed in a stereotactic frame (RWD Life Science, China), and injected with a total of 1 × 10^5^ U87GICs-LUC in 10 μl of PBS using a 27-gauge needle at 2 mm lateral and posterior to the bregma and 3 mm below the dura. Tumor dimensions were measured every week and animals were sacrificed 5 weeks after inoculation. Cell suspension was injected slowly in 20 min. Then the needle was kept in the injection site for 5 min before removing it. Tumor cells bioluminescence imaging was performed to assess xenograft formation at 7 days after implantation by using the IVIS Spectrum Live Imaging System.

### Statistical analysis

All quantified data represent an average of at least three experiments unless otherwise indicated, and standard deviations were calculated. All statistical analyses were performed using GraphPad Prism 6.0 (GraphPad Software, La Jolla, CA, USA). Comparisons among groups were performed using unpaired Student’s t-tests. Statistical significance was designated as follows: *: *p* value < 0.05, **: *p* value < 0.01, ***: *p* value < 0.001.

## Results

### Increased expression of Notch1 was detected in GBM tissues

**Fig. 1:**
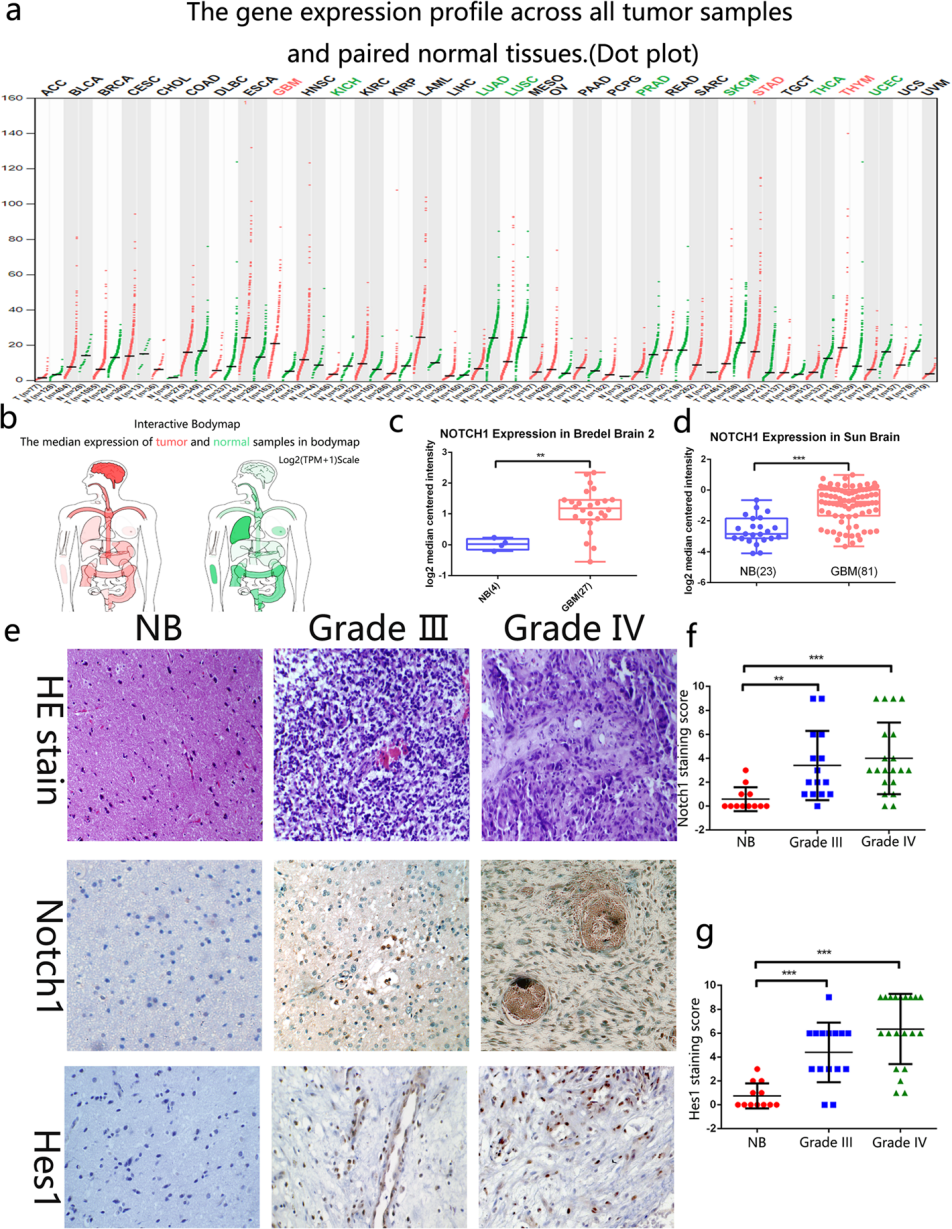
Notch1 is highly expressed in glioblastoma patients. **a** and **b** Bar plot and interactive bodymap of Notch1 expression levels across all tumor samples. (The extension of tumor abbreviations is available in Additional file 4: Table S1) (**c** and **d**) The Notch1 expression in normal brain tissue and GBM across two datasets. (*: *P* < 0.05, **: *P* < 0.01, ***: *P* < 0.001) (**e**) The Immunohistochemical staining of Notch1 signaling protein (Notch1 and Hes1) in control brain tissues and high grade glioma tissues respectively. (**f-g**) The quantitative score staining scores were shown. (*P < 0.05, **P < 0.01, ***P < 0.001)

To determine the expression of Notch1 pattern in GBM patients. Firstly, we compared the Notch1 mRNA expression profile across all tumor samples and paired normal tissues in GEPIA (http://gepia.cancer-pku.cn). Compared to other tumors, Notch1 in GBM has a much higher Tumor/Normal tissues ratio (Fig. 1a, b). In Sun Brain and Bredel Brain2 database, Notch1 expression was significantly higher in GBM compared to that in normal brain tissues (NBT) (Fig. 1c, d). Furthermore, immunohistochemical (IHC) staining of Notch1 and Hes1 proteins were performed on 35 paraffin-embedded high grade glioma samples (15 cases anaplastic astrocytoma and 20 cases GBM) and 12 control brain samples. The Notch1 signaling pathway proteins (Notch1, Hes1) were obviously elevated in high grade glioma tissues particularly in GBM tissues (Fig. 1e). Based on this result, we calculated the immunoreactive scores of the different tissue specimens (Fig. 1f and g). Western blot analysis of different grades of glioma tissues also demonstrated that Notch1 is highly expressed in high grade gliomas (WHO III-IV) (Additional file 1: Figure S1a). In addition, the expression profile of Hes1 and its Kaplan-Meier analysis also confirmed this finding (Additional file 2: Figure S2a-e). Meanwhile, a western blot assay was used to measure the Notch1 expression in GBM cell lines, the results revealed that Notch1 was commonly expressed in GBM cell lines (LN-18, SNB-19, U87, U251, LNZ308), highly expressed in the U87, U251 and LN-18 cell lines (Additional file 1: Figure S1b).

### Notch1 and CXCR4 are enriched in GICs and co-expressed with stemness markers in GBM tissues

**Fig. 2:**
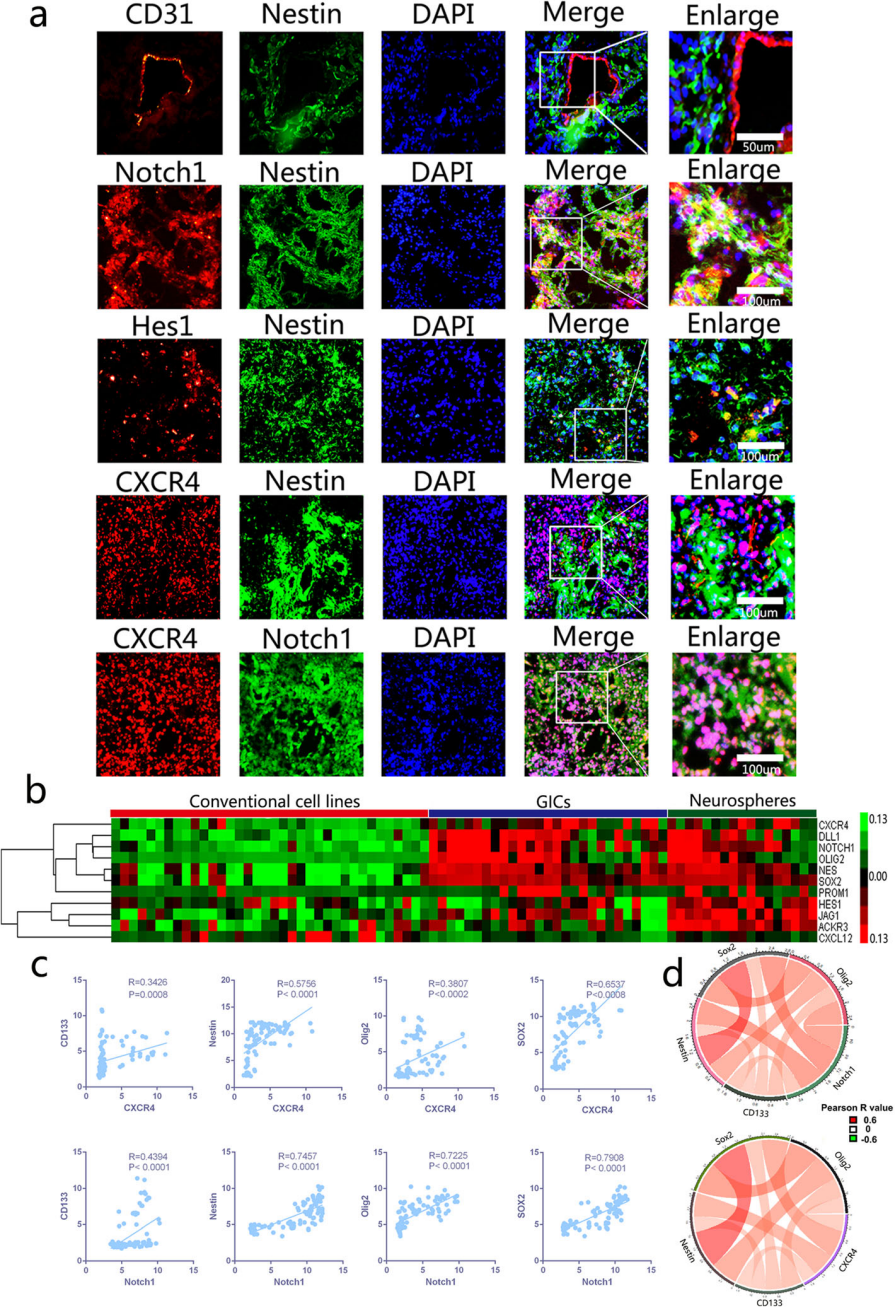
Notch1 and CXCR4 are co-expressed in GBM tissues and enriched in GICs. **a** Co-immunofluorescent staining of GIC marker Nestin (in green) with CD31, Notch1, Hes1 and CXCR4 (in red) in human GBMs. Co-immunofluorescent staining of Notch1 (in green) and CXCR4 (in red) in human GBMs. **b** Expression heatmap of interest genes in GICs and Neurospheres relative to conventional cell lines from GEO profile (NCBI GEO datasets: GSE23806). **c** Pearson analysis showing a positive correlation of GIC markers with Notch1 and CXCR4 respectively from GSE23806 dataset. **d** Circos plots demonstrate the results of Pearson analysis

To determine the relationship between Notch1 and CXCR4 in GBMs, firstly, we detected the expression of CXCR4 proteins in glioma tissues by immunohistochemical staining and western bloting, the results demonstrated that CXCR4 are incresingly expressed in gliomas according to tumor grades and the highest expression levels were detected in GBMs (Additional file 1: Figure S1a and c). Besides, tissue immunofluorescent (IF) staining was performed to observe the distribution of Notch1 signaling molecular and CXCR4 in GBM clinical specimens at a protein level, we found that Nestin (a neural stem cell marker) was expressed in tumor cells adjacent to of the blood micro-vessels labeled by CD31. Furthermore, Notch1, Hes1 and CXCR4 expressing cells colocalized with or adjacent to the Nestin expressing glioma cells. CXCR4 and Notch1 are also colocalized in same cells within GBM tissue. (Fig. 2a). Then, we retrieved data (NCBI GEO dataset: GSE23806) on the expression of Notch1 signaling molecular and CXCR4 in 36 conventional cell lines, 27 glioma stem like cell lines and 17 glioblastoma derived neurospheres. We compared the expression of the interest pathway molecules in the cell lines and found that the expression of Notch1 signaling genes and CXCR4 was significantly higher in glioma stem like cells and neurospheres than in conventional cell lines (Fig. 2b). Moreover, we examined Notch1 and CXCR4 with stemness markers in GSE23806 dataset using Pearson correlation assay (Fig. 2c-d). Both Notch1 and CXCR4 showed high correlation with stemness markers among GBM cell lines, suggesting synergistic effects in stem maintenance.

### Notch1 and CXCR4 presented a discrepant expression pattern in GICs

**Fig. 3:**
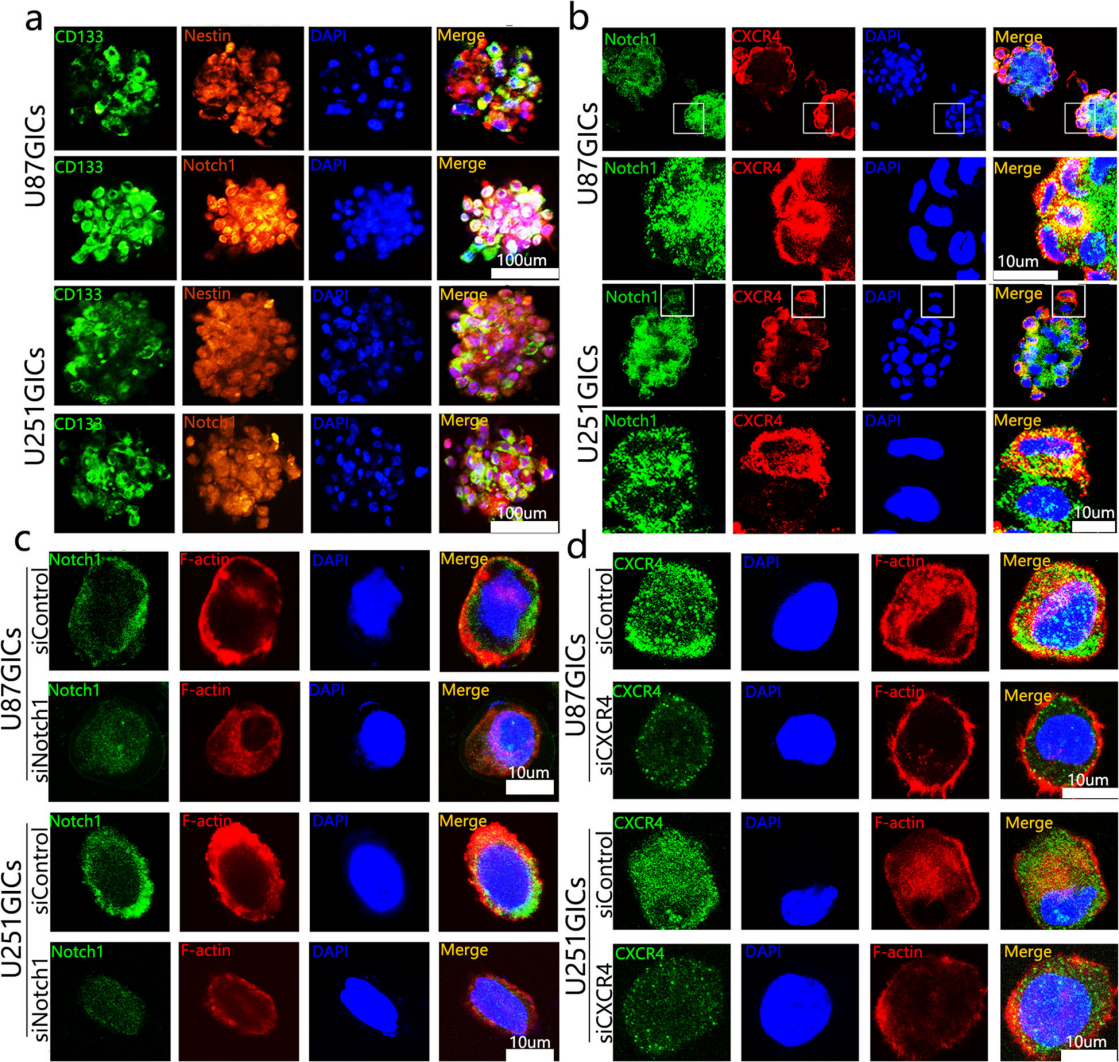
Notch1 and CXCR4 presented discrepant expression patterns in GICs. **a** Co-immunofluorescent staining of CD133 and Nestin was performed to assess GIC phenotype, CD133 and Notch1 staining was used to identify Notch1 signaling activity in GIC tumorspheres. **b** Co-immunofluorescent staining of Notch1 and CXCR4 in GIC tumorspheres. **c** Co-immunofluorescent staining of Notch1 and F-actin **d** CXCR4 and F-actin. Images were captured by laser confocal microscope

To study the regulatory mechanism of the Notch1 pathway and CXCR4 in GICs, we applied Magnetic Activated Cell Sorting (MACS) to enrich CD133+ cells from U87 and U251 glioma cells, both CD133 and Nestin were stained to assess the stem cell phenotype, CD133 positive cells exhibited a high Notch1 activity (Fig. 3a). Not quietly consistent with our speculation, confocal microscopy immunofluorescence of glioma tumorsphere displayed that contrasting to Notch1 positive glioma cells which comprised a large proportion in the CD133+ glioma cell spheres, CXCR4 positive glioma cells only represent a small subset of CD133+ glioma cell spheres. Interestingly, these CXCR4 positive glioma cell, usually expressed Notch1 both and dispersed in the periphery of the sphere (Fig. 3b). Furthermore, U87/U251 GICs transfected with siControl and siCXCR4 were subjected to F-actin staining. We observed that the CXCR4 protein was almost co-localized with F-actin, meanwhile, the decreased CXCR4 expression paralleled with a weaker microfilament staining (Fig. 3d). However, these phenomena were not observed in siControl and siNotch1 U87 and U251 GICs (Fig. 3c). As the cytoskeleton F-actin was considered to be bounded up with various cancer biology mainly includes cell migration and invasion as well as in self-renewal [7, 26, 27], CXCR4 might exert a crucial role in stem maintenance and migration of GICs.

### Notch1 contributes to the invasion, migration and self-renewal of GICs by CXCL12/CXCR4 system

**Fig. 4:**
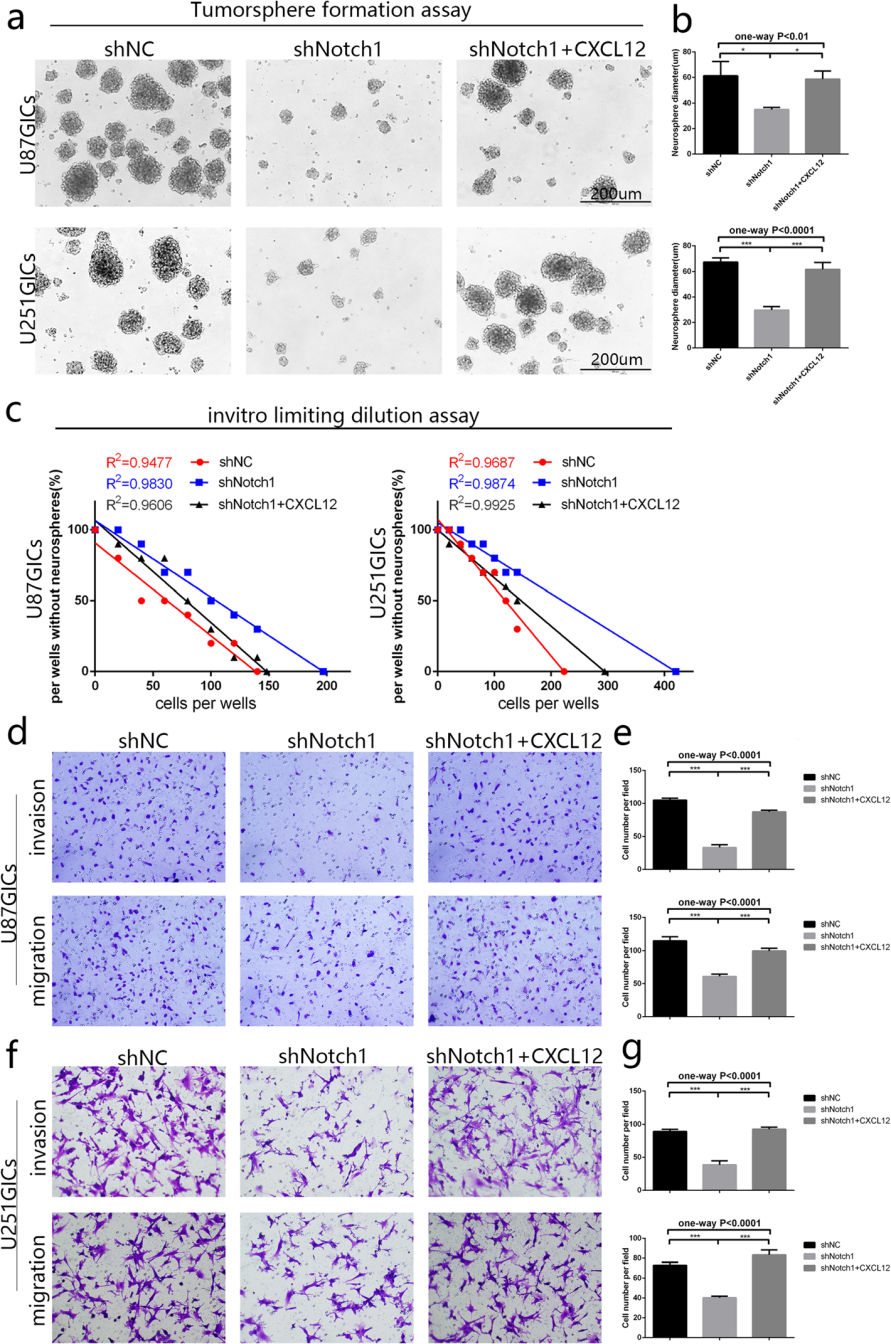
Notch1 is required for invasion, migration properties and self-renewal of GICs by CXCL12/CXCR4 system. **a-b** Morphology of tumor spheres formed by the cancer initiating cells from U87 and U251 glioma cells. And Quantitative analysis were shown respectively. **c** In vitro limiting dilution assay of U87GICs and U251GICs in negative control, shRNA-Notch1 and shNotch1 + CXCL12 groups. **d-g** Transwell assay of U87GICs and U251GICs in negative control, shRNA-Notch1 and shNotch1 + CXCL12 groups. And Quantitative analysis were shown respectively. (*P < 0.05, **P < 0.01, ***P < 0.001)

The presence of GICs has been reported in the U87 and U251 cell [28] lines as well as the fact that Notch1 could maintain glioma cell stemness [7, 29, 30]. To investigate the mechanisms underlying Notch1 regulation of GICs invasion and self-renewal, stable Notch1-knockdown glioma initiation cells (U87-shNotch1 and U251-shNotch1) were established (Additional file 3: Figure S3a-b) and western blot showed that inhibition of Notch1 pathway through shRNA or MK0752 in U87 and U251 derived GICs could quantitatively decrease the expression of CXCR4, but the expression of CXCL12 made no difference in the shNC and shNotch1 groups (Additional file 3: Figure S3c-d). To examine the effects of Notch1 on the self-renewal properties of GICs, tumorsphere formation and limiting dilution assay was performed. We observed a significant decreased volume and a higher number of cells required to generate tumor sphere in shNotch1 group while the additional 100 ng/ml of CXCL12 could significantly rescue this trend and boost the self-renewal ability of GICs (Fig. 4a-c). To investigate the effects of Notch1 on invasion and migration ability in GICs, Transwell invasion and migration assays was performed and revealed that downregulation of Notch1 could significantly restrained both migration and invasion in U87GICs and U251GICs. Moreover, addition of CXCL12 (100 ng/ml) partly rescued downregulation of Notch1 mediated cell migration and invasion restriction (Fig. 4d-g). Those results demonstrate that Notch1 contributes to the invasion, migration and self-renewal of GICs through CXCL12/CXCR4 system.

### Notch1 potentiates the AKT/m-TOR pathway and CXCL12/CXCR4 axis in GICs

**Fig. 5:**
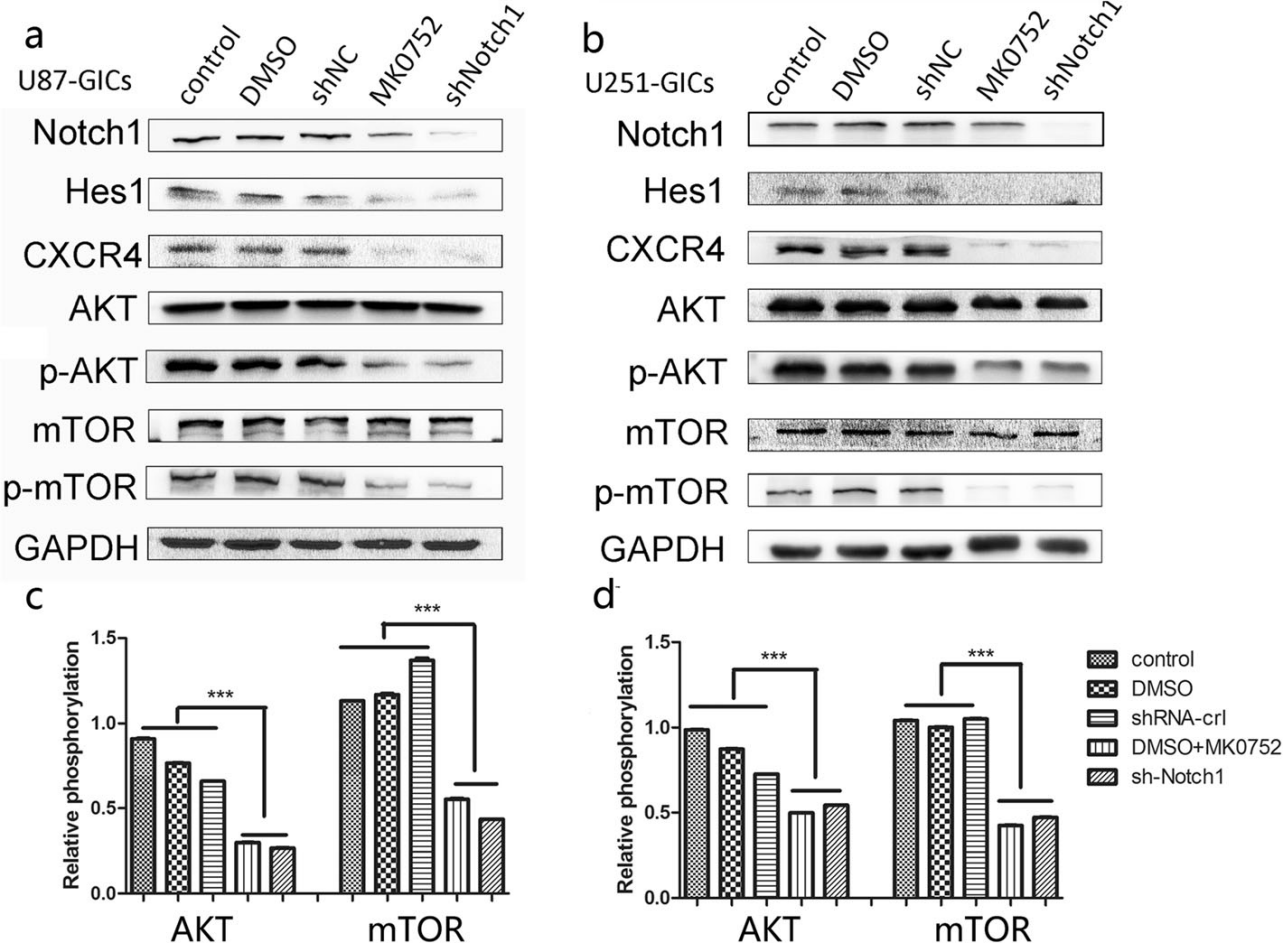
Notch1 enhance the activity of AKT/mTOR pathway and CXCL12/CXCR4 axis. **a-b** Decreased protein level of Hes1 (Notch1 pathway downstream factors), CXCR4, p-AKT and p-mTOR were measured by western blot after blockage of Notch1 signaling by shRNA-Notch1 and MK0752 in U87GICs and U251GICs. **c-d** The relative phosphorylation of AKT and mTOR were shown respectively. (*P < 0.05, **P < 0.01, ***P < 0.001)

It has been reported that activating the Notch1/Hes1 pathway could accelerate cell invasion, migration and proliferation through the PI3k/AKT/mTOR pathway in acute T lymphocyte leukaemia and mesothelioma [31, 32]. The Notch pathway has also been found to promote ovarian cancer growth and migration via the CXCL12/CXCR4 chemokine system [33]. However, in GICs, the exact mechanism has not been elucidated. MK-0752 is a potent γ-secretase inhibitor (GSI), which could block the Notch intracellular domain (NICD) and the expression of Hes1. To further confirm that Notch1 promotes invasion and self-renewal and tumor growth by potentiating the AKT pathway, MK0752 was used to inhibit the Notch1 pathway. To determine the optimal concentration of MK0752, U87GICs were treated with different concentrations of MK0752 (0, 10, 20, 40, 80, 100, 150 nM) for 48 h, respectively. The results showed that MK0752 treatment reduced the protein expression of activated Notch1 (NICD) in a dose-dependent manner (Additional file 3: Figure S3e). Due to no significant differences between the 80 and 100 nM of MK0752 treatments via western blot, 80 nM of MK0752 was used in the subsequent experiments. The Notch1 pathway was downregulated with shRNA or MK0752 among U87GICs and U251GICs. The expression of CXCR4 and the phosphorylation levels of AKT and mTOR were significantly inhibited (Fig. 5a-d).

### Blockage of Notch1 attenuates AKT/mTOR signaling activity by CXCL12/CXCR4 system

**Fig. 6:**
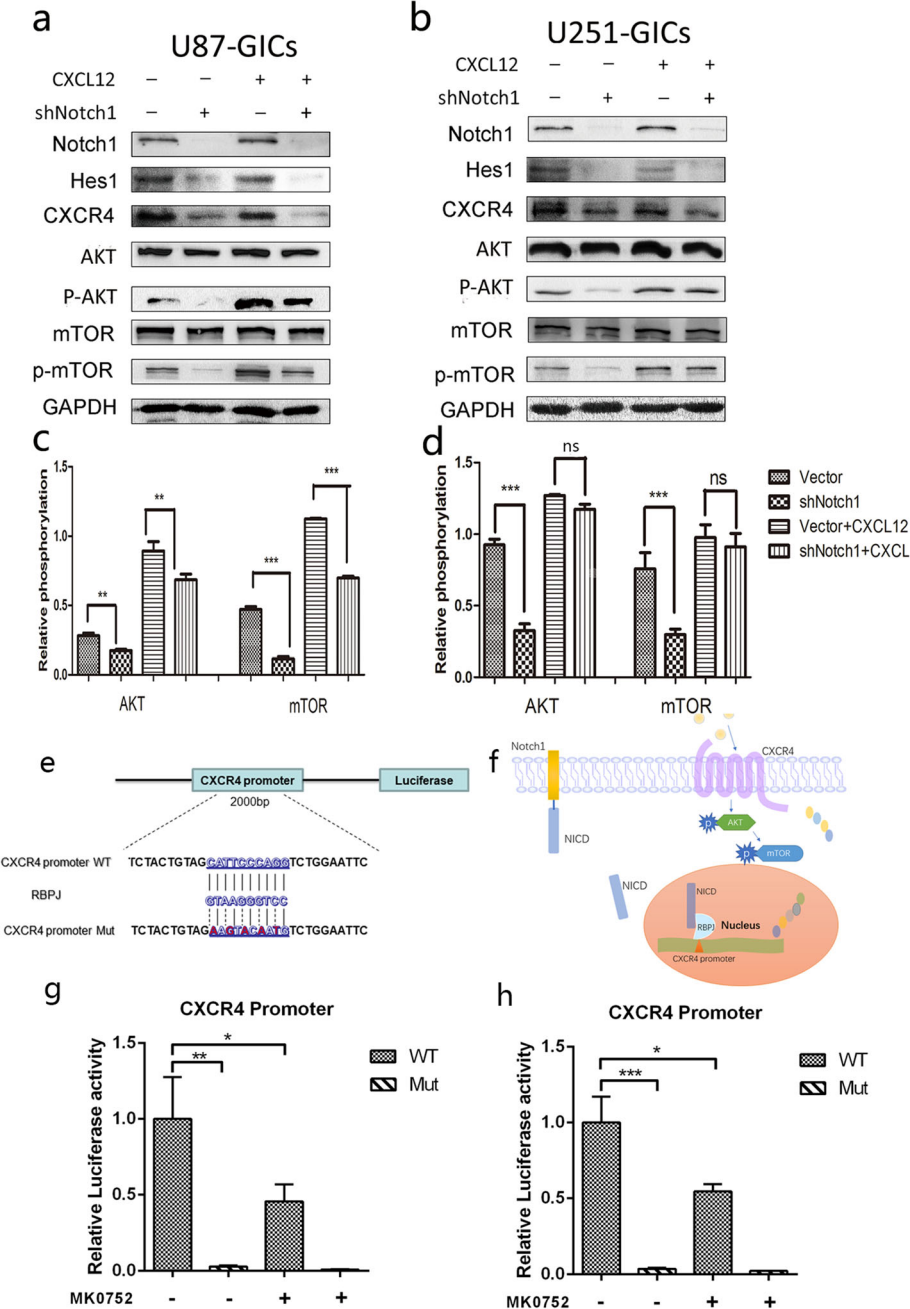
Notch1 enhance the activity of AKT/mTOR pathway through CXCL12 /CXCR4 axis. **a** and **c** The representative blot and relative phosphorylation of AKT and mTOR in U87GICs were shown. **b** and **d** The representative blot and relative phosphorylation of AKT and mTOR in U251GICs were shown. (*P < 0.05, **P < 0.01, ***P < 0.001, ns: no significance) **e** Schematic representation of the potential binding sites for RBPJ in CXCR4 promoter. and mutant type of luciferase reporter constructs have intact and mutated seed sequences. **g** and **h** Dual-reporter luciferase assays showed the relative luciferase activity of WT and Mut reporter constructs as well as Notch1 inhibition (MK0752) group. **f** Proposed model illustrates the underlying crosstalk between Notch1 and CXCR4 in GICs through PI3k/AKT/mTOR pathway. Notch1 signaling could interact with CXCL12/CXCR4 Chemotaxis system by directly regulating CXCR4’s promoter

CXCL12 is a specific ligand for CXCR4, to demonstrate that CXCR4 is a mediator between Notch1 and the AKT/mTOR pathway, 100 ng/ml CXCL12 was used to stimulate CXCR4 after incubation for 30 min [34]. As expected, CXCL12 significantly increased AKT and mTOR phosphorylation in GICs compared to that in the cells without CXCL12 treatment. Interestingly, in the Notch1 downregulated GICs, treatment with CXCL12 resulted in no remarkable decrease in the phosphorylation of AKT/mTOR (Fig. 6a-d), which indicated that CXCL12 may reverse the downregulation of AKT/mTOR phosphorylation induced by shNotch1 and revealed that the CXCL12/CXCR4 axis is upstream of the AKT/mTOR. We further investigated the regulatory mechanism underlying the correlation between Notch1 and CXCR4 by dual-luciferase reporter assays, RBPJ, the downstream transcription factor of Notch1 signaling was proposed to bind to the region of the CXCR4 using PROMO tool (Fig. 6e). Our results indicated that inhibition of Notch1 signaling or mutated RBPJ binding area of CXCR4 promoter could significantly reduce the CXCR4 promoter activity in U87 and U251GICs (Fig. 6g and h), suggesting that RBPJ directly binds to the CXCR4 promoter to control its transcription thus modulating AKT/mTOR activity (Fig. 6f).

### Silencing of Notch1 inhibits tumor growth and invasion in vivo

**Fig. 7:**
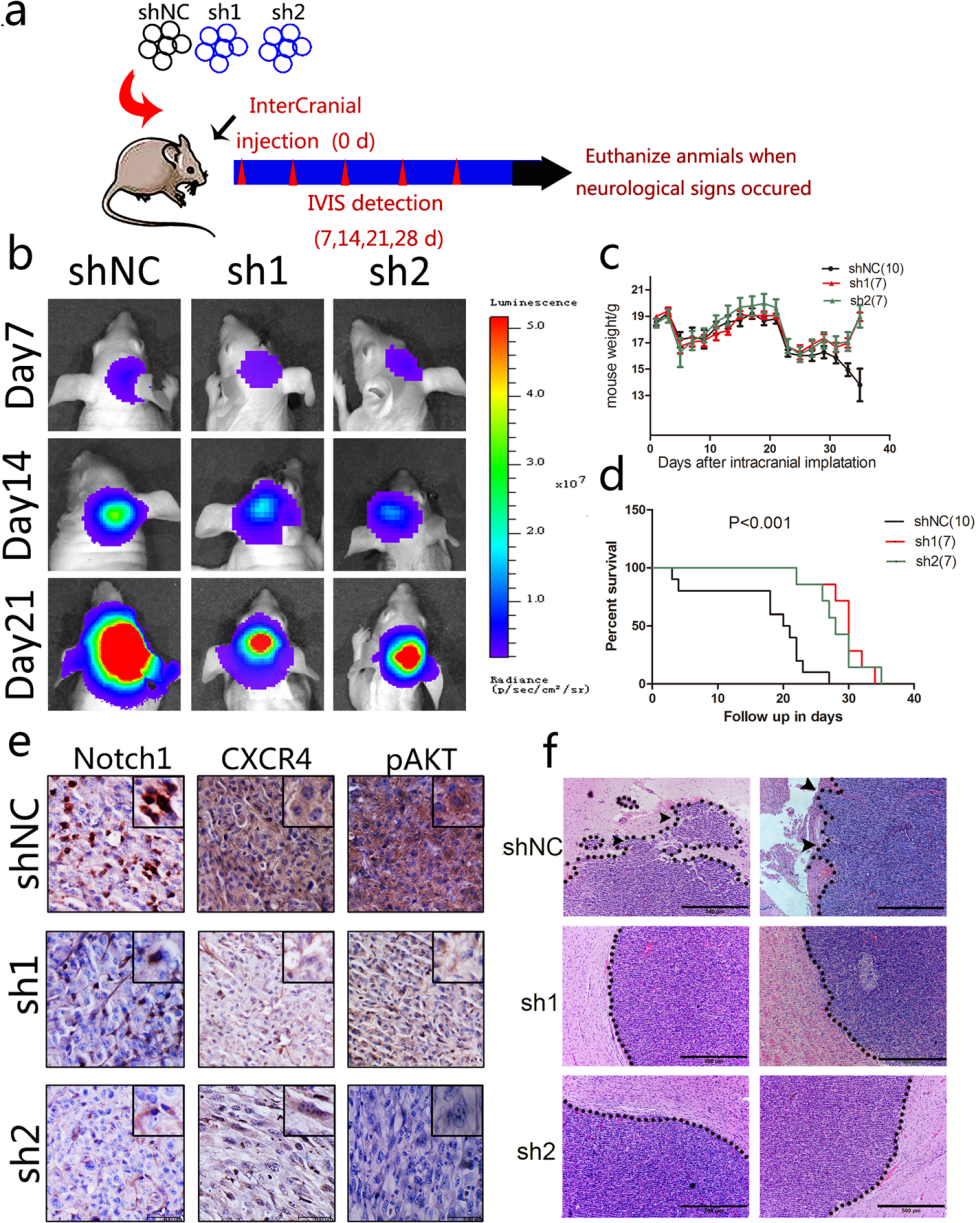
Silencing of Notch1 inhibits tumor growth and invasion in vivo. **a** Schematic diagram of Intracranial implantation of U87-GICs expressing shNotch1 or shNC. **b** The representative bioluminescent images of the tumor-bearing mice implanted with U87-GICs. **c** Mouse weight was recorded as a measure of mouse nutrition in the two groups. **d** Kaplan–Meier survival curve was used to detect differences in mouse survival times between the two groups. **e** Immunohistochemistry analysis of the expression of Notch1, CXCR4 and pAKT in Notch1-shRNA treated tumors compared to tumors in the shNC group. **f** Representative images of H&E staining of tumor border from mice 3 weeks after tumor implantation (black triangle: disseminated tumors)

To investigate the influence of Notch1 expression on tumor growth in vivo, we performed experiments according to the schematic diagram (Fig. 7a). We observed shRNA-NC or shRNA-Notch1 GICs in mouse brains in vivo by living imaging which showed increasing radiance values corresponding to increasing tumor growth. We found that the radiance values were lower in shNotch1 tumors compared with control group (Fig. 7b). Furthermore, the hematoxylin and eosin (HE) stained intracranial tumors also showed smoother tumor borders in the shNotch1 groups (Fig. 7f). Likewise, Notch1 downregulation was associated with a longer survival (Fig. 7d) and a decreased weight loss in mice (Fig. 7c). In addition, Immunohistochemistry (IHC) displayed decreased expression levels of Notch1, CXCR4 and pAKT in shNotch1 group, the result consistent with in vitro experiments (Fig. 7e).

## Discussion

As early as 2006, Androutsellis-Theotokis et al. demonstrated that Notch signaling could regulate stem cell numbers in vitro and in vivo [35]. Since then, numerous studies have been carried out. Up to now, Notch signaling has been found to be involved in many cell fate decisions such as cell proliferation, differentiation and apoptosis during normal development and in the genesis of several cancers. Most recently, several studies have reported that Notch was enriched in CSCs and has been shown to maintain the stem cell phenotype [7, 36]. In gliomas, Notch seems to confer radio-resistance to cancer stem cells [11], and inhibition of Notch signaling through γ secretase inhibitors could decreased the tumor neurospheres growth [35], glioblastoma xenograft initiation and clonogenic growth in vivo [37]. Although Notch1 plays a vital role in a variety of tumors, most of the clinical trials of Notch1 have showed modest efficacy and only in the initial stage, it is probably due to the complex heterogeneity of glioma and the crosstalk between Notch pathway and other molecules. Consequently, it is urgent to understand the mechanism of Notch regulation of tumorigenesis in GICs. Many studies have shown that Notch1 can regulate PI3K /AKT/mTOR pathway activity. In T-cell acute lymphoblastic leukemia (T-ALL), Teresa et al. revealed that Hes1 could decrease the protein level of PTEN and enhance the activity of the PI3K/AKT/mTOR pathway [38]. Shepherd et al. found that drugs targeting PI3K/mTOR can upregulate NOTCH-MYC activity leading to a cytotoxic response [39]. In addition to PTEN, the Notch pathway also regulates PI3K/AKT/mTOR pathway activity via P53 and EGFR [40, 41]. However, the biological behavior and related molecular mechanism of Notch1 in glioma initiating cells are not well reported worldwide.

A growing number of studies have found that CXCR4 is more likely present within the glioma stem like cell population compared with differentiated glioma cells. It is increasingly expressed in glioma cells corresponding to increasing tumor grade [42]. There have been various reports suggesting that CXCR4 is required for tumor proliferation, invasion, angiogenesis, and modulation of the immune response [14]. It may also serve as a prognostic factor in characterizing subsets of glioblastoma multiforme [42], as patients with CXCR4-positive gliomas seem to have a worse prognosis after surgery and is associated with an angiogenic switch in recurrence of glioblastoma after radio-chemotherapy [43, 44]. Ping et al. [34] found that compared with CD133- cells, CD133+ GSCs expressed significantly higher levels of CXCR4, and migrated more efficiently in response to the CXCR4 ligand CXCL12. Liang [45] reported that the SDF-1/CXCR4 axis can promote the phosphorylation of AKT and then increase the expression of VEGF and promote angiogenesis in breast cancer. The few studies that have looked into the role of CXCR4 in Notch1 signaling among several types of cells. In mesenchymal stem cells, for example, mRNA levels of CXCR4 were significantly increased when Notch signaling was interrupted by c-secretase inhibitor (GSI) or knockout of the transcription factor RBP-J [46]. But the same treatment, down-regulates CXCR4 expression in ovarian cancer [33], suggesting complicated relationship between Notch signaling and the CXCR4 expression.

**Fig. 8:**
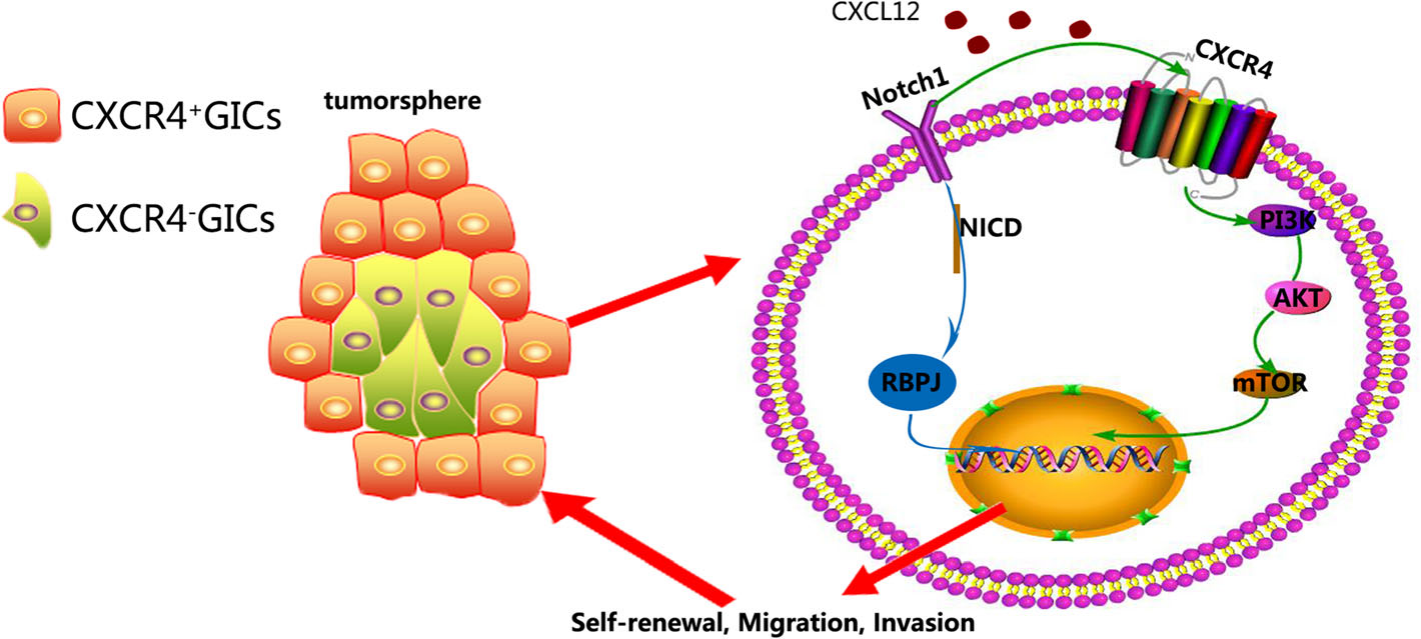
Model of the mechanism of Notch1 signaling and CXCL12/CXCR4 axis in GICs self-renewal, invasion and tumor growth. CXCR4 positive glioma cells, usually expressed Notch1 both and dispersed in the periphery of the tumorsphere. Notch1 signaling promotes the self-renewal, invasion and tumor growth of glioma initiating cells via AKT/mTOR pathway activity medicated by CXCL12/CXCR4 axis

In this study, we provide evidence that Notch1 signaling is highly activated in GBM tissues and its target gene Hes1 is correlated with poor patient survival. Moreover, our results showed that Notch1 signaling and CXCR4 were highly correlated in GBM tissues and enriched in GICs. However, in GICs, Notch1 and CXCR4 presented a discrepant expression pattern. As the CXCR4 positive glioma initiating cells co-expressed with Notch1 usually dispersed in the periphery of the tumorsphere, we put forward the hypothesis that CXCR4 might exert a crucial role in the Notch1 signaling mediated stem maintenance and migration of GICs. Subsequently, our study demonstrated that Notch1 downregulation could significantly inhibit glioma initiating cell biological behavior including self-renewal invasion and migration in vitro, as well as tumor growth in vivo. Furthermore, western blot analysis and luciferase reporter assay were employed to clarify the potential mechanism involved in Notch1 regulated GICs, we found that CXCL12 / CXCR4 acted as a central node between Notch1 signaling and AKT/mTOR pathway. Notch1 signaling could modulate CXCR4 through directly targeting its promoter (Fig. 8).

## Conclusions

Our findings provide direct evidence that Notch1 pathway promotes GIC self-renewal, invasion and tumor growth through PI3K pathway activity via upregulating CXCR4 expression. Furthermore, the CXCL12/CXCR4 axis is an important immediate effector of Notch1 that mediates downstream mechanisms. All of these findings would facilitate the development of molecular therapies targeting the Notch1 signaling pathway for GBM management.

## Additional files

Additional file 1: **Figure S1.** The expression pattern of Notch1 and CXCR4 in glioma tissues and cell lines. (a) The protein expression of Notch1 and CXCR4 in different grade glioma tissues. (b) The expression of Notch1 in GBM cell lines. (c) The expression of CXCR4 and CXCL12 in high grade glioma tissues through immunohistochemistry analysis. (TIF 7560 kb)

Additional file 2: **Figure S2.** The expression of Hes1 and its association with patient’s prognosis. (a-c) Accordingly, in contrast to NBT or low-grade astrocytoma, Hes1 (Notch1 targeting gene) was notably upregulated in GBM patients according to TCGA, Sun Brain and French’s datasets. (d) Kaplan-Meier analysis of overall survival probability for the TCGA database with the log rank test *P* value was indicated. (e) Kaplan-Meier analysis of overall survival probability for the CGGA database with the log rank test *P* value indicated. (TIF 4670 kb)

Additional file 3: **Figure S3.** Downregulation of Notch1 signaling through pharmacological intervention and RNA interference. (a) Knockdown of Notch1 in two RNA interference sequence, and the shRNA with a better effect was used in the invitro study. (b)Western blot assay was used to characterize the expression of Notch1 signaling protein in shNotch1 U87GICs and U251GICs. (c) Reduction of NICD and CXCR4 in GICs induced by MK0752 in a dose-dependent fashion. (d) The expression of CXCL12 /CXCR4 system in the shNC and shNotch1 groups. (e) Reduction of NICD in GICs induced by MK0752 in a dose-dependent fashion (due to no significant differences in NICD expression between 80 and 100 nM of MK0752 treatment, 80 nM of MK0752 was used in the western blot assay). (TIF 3290 kb)

Additional file 4: **Table S1.** The extension of tumor abbreviations. (DOCX 17 kb) (TIF 9945 kb)

## Data Availability

All data and materials can be provided upon request.

## Abbreviation

CCLs: Conventional cell lines
ECM: Extracellular matrix
GBM: Glioblastoma multiforme
GICs: Glioma initiating cells
GSCs: Glioma stem cells
GSI: γ-Secretase inhibitor
GSLCs: Glioblastoma stem-like cell lines
HGG: High grade glioma
IF: Immunofluorescence
MACS: Magnetic activated cell sorting
NBT: Normal brain tissues
NICD: Notch intracellular domain
SDF1: Chemokine stromal cell-derived factor 1
SFM: Serum-free medium
T-ALL: T-cell acute lymphoblastic leukaemia
