# Supplementary figures and images for: Notch1 signaling pathway promotes invasion, self-renewal and growth of glioma initiating cells via modulating chemokine system CXCL12/CXCR4
Source: J Exp Clin Cancer Res. 2019 Aug 5;38:339. doi: 10.1186/s13046-019-1319-4 (PMC6683584; doi:10.1186/s13046-019-1319-4)

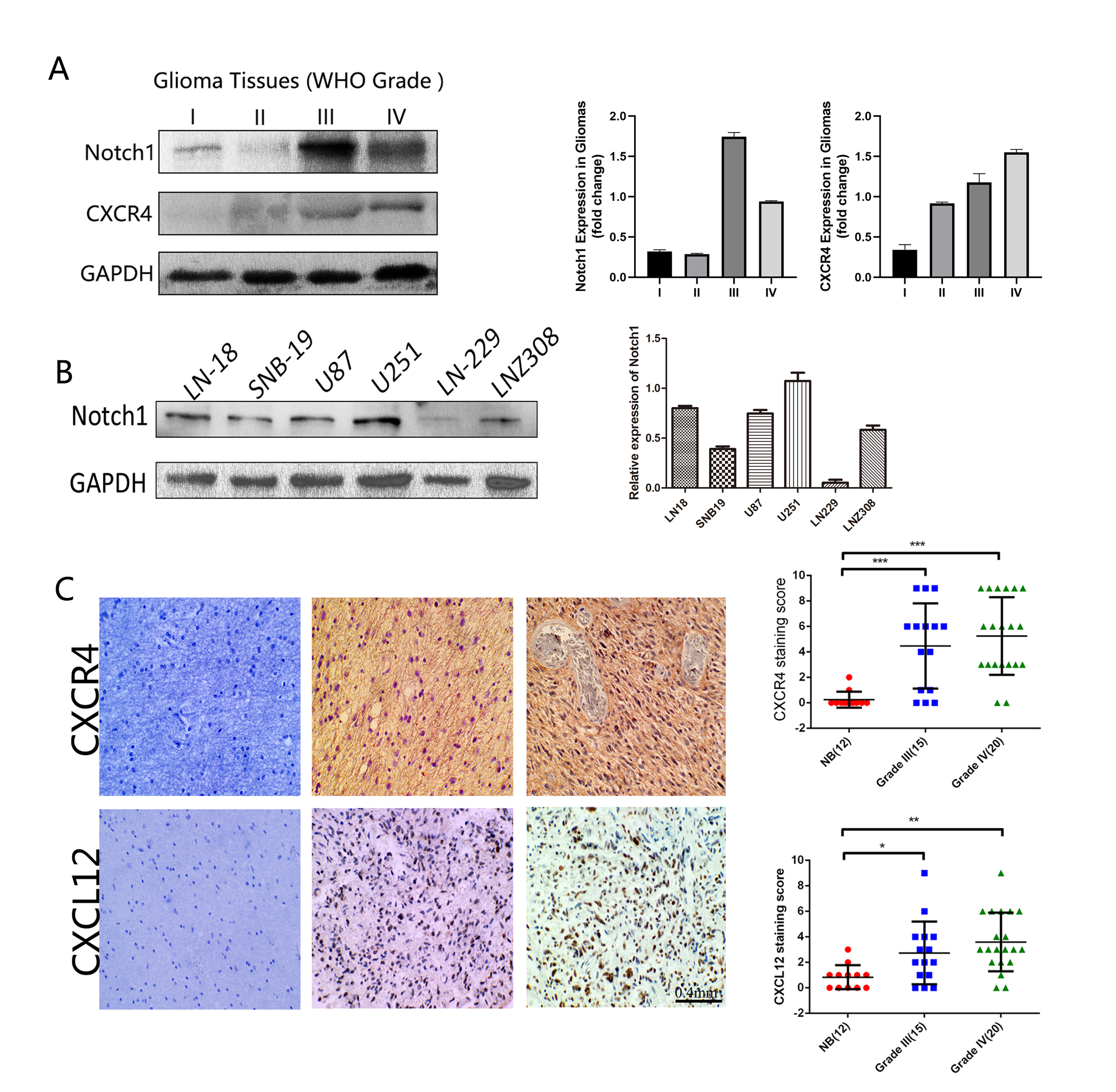

Supplement: Supplementary file 1 — Figure S1. The expression pattern of Notch1 and CXCR4 in glioma tissues and cell lines. (a) The protein expression of Notch1 and CXCR4 in different grade glioma tissues. (b) The expression of Notch1 in GBM cell lines. (c) The expression of CXCR4 and CXCL12 in high grade glioma tissues through immunohistochemistry analysis. (TIF 7560 kb) [file 13046_2019_1319_MOESM1_ESM.tif]

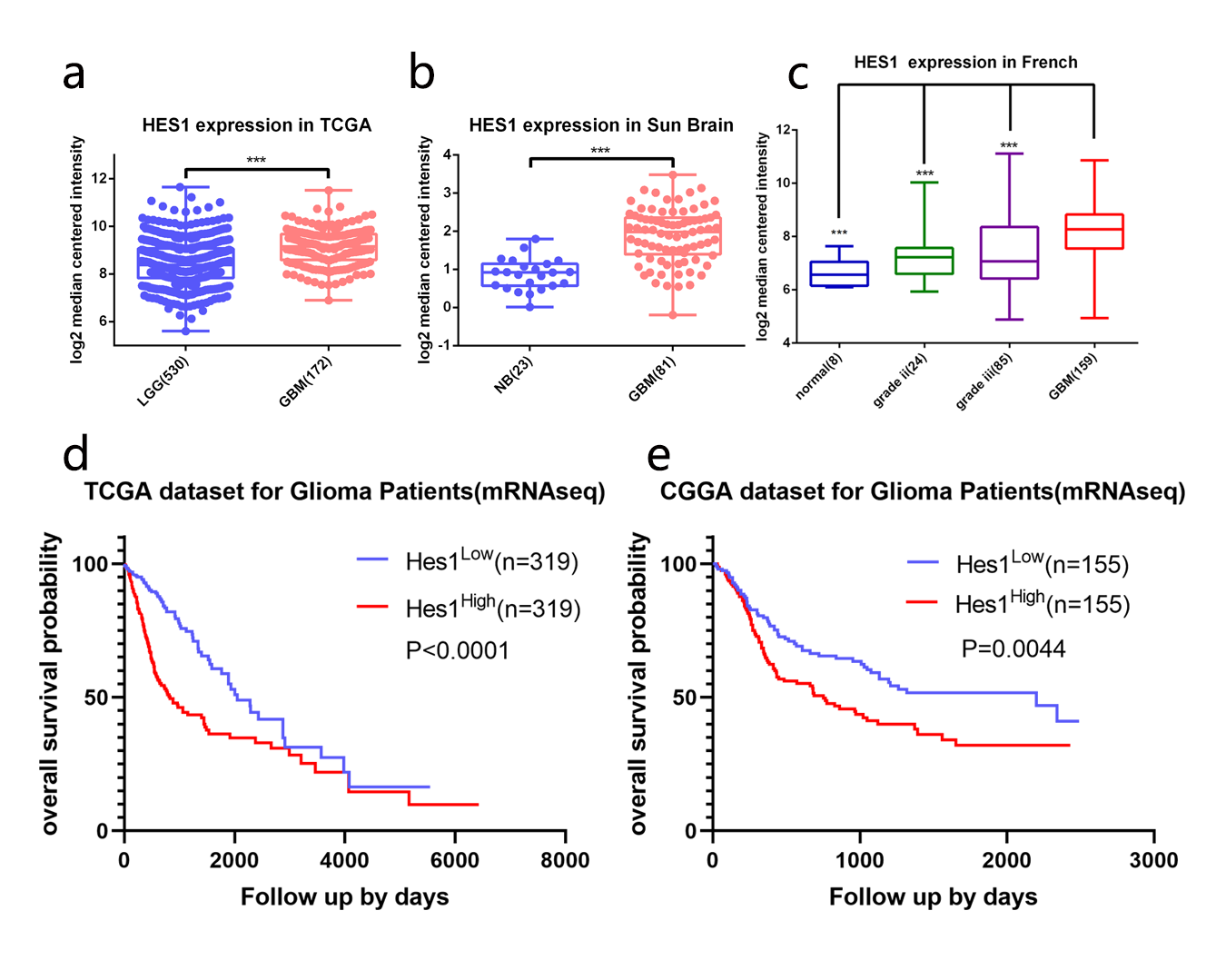

Supplement: Supplementary file 2 — Figure S2. The expression of Hes1 and its association with patient’s prognosis. (a-c) Accordingly, in contrast to NBT or low-grade astrocytoma, Hes1 (Notch1 targeting gene) was notably upregulated in GBM patients according to TCGA, Sun Brain and French’s datasets. (d) Kaplan-Meier analysis of overall survival probability for the TCGA database with the log rank test P value was indicated. (e) Kaplan-Meier analysis of overall survival probability for the CGGA database with the log rank test P value indicated. (TIF 4670 kb) [file 13046_2019_1319_MOESM2_ESM.tif]

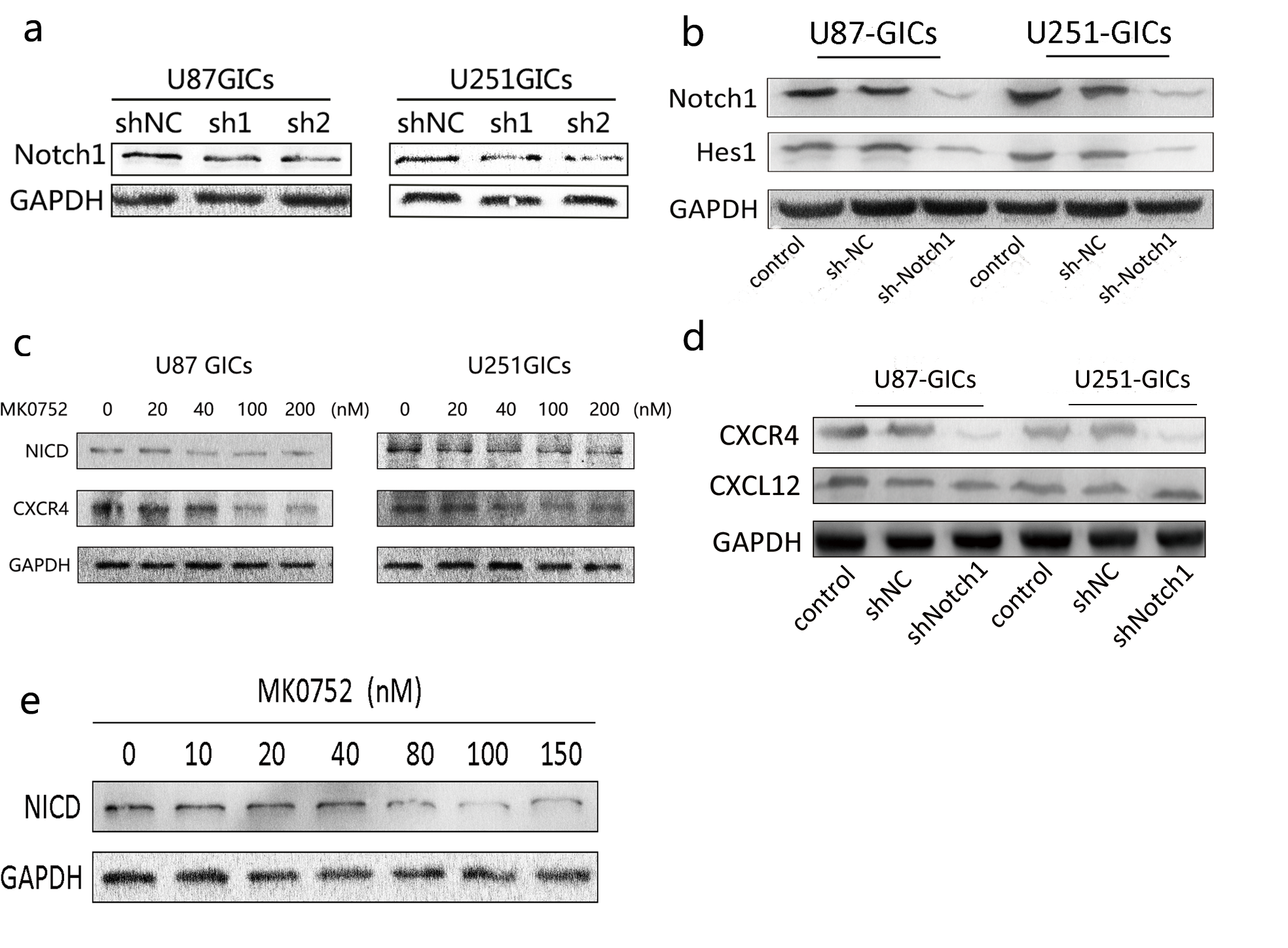

Supplement: Supplementary file 3 — Figure S3. Downregulation of Notch1 signaling through pharmacological intervention and RNA interference. (a) Knockdown of Notch1 in two RNA interference sequence, and the shRNA with a better effect was used in the invitro study. (b)Western blot assay was used to characterize the expression of Notch1 signaling protein in shNotch1 U87GICs and U251GICs. (c) Reduction of NICD and CXCR4 in GICs induced by MK0752 in a dose-dependent fashion. (d) The expression of CXCL12 /CXCR4 system in the shNC and shNotch1 groups. (e) Reduction of NICD in GICs induced by MK0752 in a dose-dependent fashion (due to no significant differences in NICD expression between 80 and 100 nM of MK0752 treatment, 80 nM of MK0752 was used in the western blot assay). (TIF 3290 kb) [file 13046_2019_1319_MOESM3_ESM.tif]

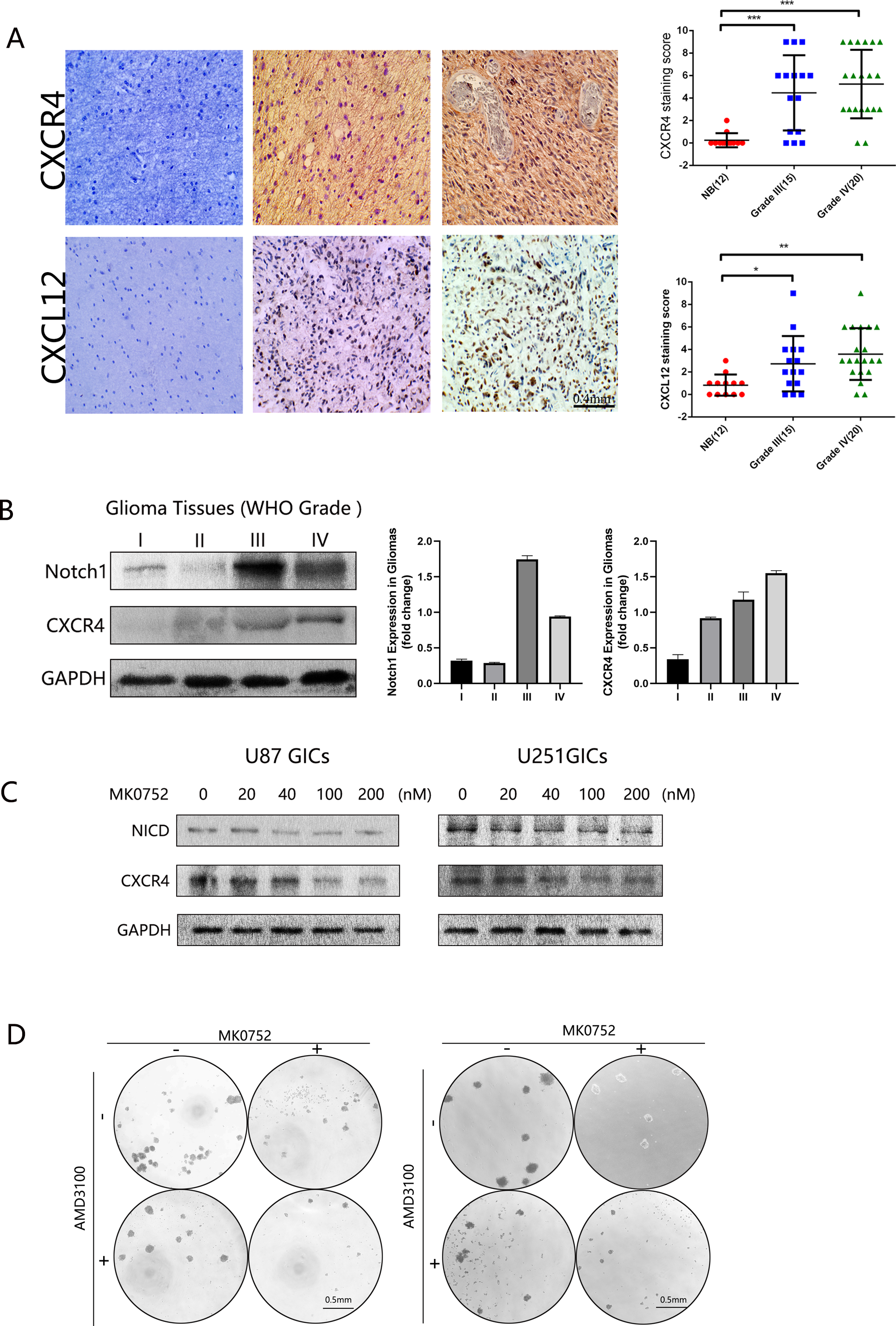

Supplement: Supplementary file 4 — Table S1. The extension of tumor abbreviations. (DOCX 17 kb) (TIF 9945 kb) [file 13046_2019_1319_MOESM4_ESM.tif]
